# μRALP and Beyond: Micro-Technologies and Systems for Robot-Assisted Endoscopic Laser Microsurgery

**DOI:** 10.3389/frobt.2021.664655

**Published:** 2021-09-08

**Authors:** Leonardo S. Mattos, Alperen Acemoglu, André Geraldes, Andrea Laborai, Andreas Schoob, Brahim Tamadazte, Brian Davies, Bruno Wacogne, Christian Pieralli, Corina Barbalata, Darwin G. Caldwell, Dennis Kundrat, Diego Pardo, Edward Grant, Francesco Mora, Giacinto Barresi, Giorgio Peretti, Jesùs Ortiz, Kanty Rabenorosoa, Laurent Tavernier, Lionel Pazart, Loris Fichera, Luca Guastini, Lüder A. Kahrs, Micky Rakotondrabe, Nicolas Andreff, Nikhil Deshpande, Olivier Gaiffe, Rupert Renevier, Sara Moccia, Sergio Lescano, Tobias Ortmaier, Veronica Penza

**Affiliations:** ^1^ Istituto Italiano di Tecnologia, Genoa, Italy; ^2^ Department of Otorhinolaryngology, Guglielmo da Saliceto Hospital, Piacenza, Italy; ^3^ Yuanda Robotics GmbH, Hannover, Germany; ^4^ Institut des Systèmes Intelligents et de Robotique, Sorbonne Université, CNRS, Paris, France; ^5^ Imperial College London, London, United Kingdom; ^6^ FEMTO-ST Institute, Univ. Bourgogne Franche-Comte, CNRS, Besançon, France; ^7^ Centre Hospitalier Régional Universitaire, Besançon, France; ^8^ Mechanical and Industrial Engineering Department, Louisiana State University, Baton Rouge, LA, United States; ^9^ Department of Electrical and Computer Engineering, North Carolina State University, Raleigh, NC, United States; ^10^ Clinica Otorinolaringoiatrica, IRCCS Policlinico San Martino, Genoa, Italy; ^11^ Dipartimento di Scienze Chirurgiche e Diagnostiche Integrate, Università Degli Studi di Genova, Genoa, Italy; ^12^ Department of Robotics Engineering, Worcester Polytechnic Institute, Worcester, MA, United States; ^13^ Department of Mathematical and Computational Sciences, University of Toronto, Mississauga, ON, Canada; ^14^ National School of Engineering in Tarbes, University of Toulouse, Tarbes, France; ^15^ The BioRobotics Institute, Scuola Superiore Sant’Anna, Pisa, Italy; ^16^ Institute of Mechatronic Systems, Leibniz Universität Hannover, Garbsen, Germany

**Keywords:** laser microsurgery, micro-robot, flexible robotic endoscope, surgeon-robot interface, cancer imaging, augmented reality, computer-assisted surgery, cognitive surgical system

## Abstract

Laser microsurgery is the current gold standard surgical technique for the treatment of selected diseases in delicate organs such as the larynx. However, the operations require large surgical expertise and dexterity, and face significant limitations imposed by available technology, such as the requirement for direct line of sight to the surgical field, restricted access, and direct manual control of the surgical instruments. To change this status quo, the European project μRALP pioneered research towards a complete redesign of current laser microsurgery systems, focusing on the development of robotic micro-technologies to enable endoscopic operations. This has fostered awareness and interest in this field, which presents a unique set of needs, requirements and constraints, leading to research and technological developments beyond μRALP and its research consortium. This paper reviews the achievements and key contributions of such research, providing an overview of the current state of the art in robot-assisted endoscopic laser microsurgery. The primary target application considered is phonomicrosurgery, which is a representative use case involving highly challenging microsurgical techniques for the treatment of glottic diseases. The paper starts by presenting the motivations and rationale for endoscopic laser microsurgery, which leads to the introduction of robotics as an enabling technology for improved surgical field accessibility, visualization and management. Then, research goals, achievements, and current state of different technologies that can build-up to an effective robotic system for endoscopic laser microsurgery are presented. This includes research in micro-robotic laser steering, flexible robotic endoscopes, augmented imaging, assistive surgeon-robot interfaces, and cognitive surgical systems. Innovations in each of these areas are shown to provide sizable progress towards more precise, safer and higher quality endoscopic laser microsurgeries. Yet, major impact is really expected from the full integration of such individual contributions into a complete clinical surgical robotic system, as illustrated in the end of this paper with a description of preliminary cadaver trials conducted with the integrated μRALP system. Overall, the contribution of this paper lays in outlining the current state of the art and open challenges in the area of robot-assisted endoscopic laser microsurgery, which has important clinical applications even beyond laryngology.

## Introduction

Lasers form an increasingly common surgical tool for precision treatment of pathological conditions on delicate and vital human organs. One example is transoral laser microsurgery (TOLMS), which involves the use of a surgical laser and challenging surgical techniques for treating abnormalities in the glottis and supraglottic regions (Steiner and Ambrosch, 2000).

TOLMS is the current gold-standard technique for phonomicrosurgeries, i.e., the surgical treatment of the vocal cords (Rubinstein and Armstrong, 2011). These are delicate operations that require high surgical precision. However, they are currently performed with very limited technological support, so large surgical dexterity and expertise is needed. The consequence is that the quality of such surgeries relies completely on the dexterity and capabilities of the operating surgeon, who must control the surgical tools with micrometric precision to both eradicate the disease and minimize damage to healthy tissue. If not performed adequately, the outcome of phonomicrosurgery can have a large impact on the quality of life of the patient, as it can affect both phonation and deglutition (Presutti, 2010).

Performing TOLMS currently requires the use of a laryngoscope to provide both adequate visualization and access to the surgical field. The laryngoscope is basically a metal tube that is inserted through the mouth of the patient to provide this required operative channel. It allows the use of an external microscope and dedicated surgical tools. The surgeon operates through the laryngoscope while using a microscope, a laser micromanipulator and long microsurgical forceps.

The ergonomics of the current TOLMS setup is also sub-optimal, complicating the achievement of high precision surgical tasks. Particularly, armrests are needed to stabilize the surgeon’s hands during the delicate control of the laser micromanipulator and of the long laryngeal forceps. In addition, other difficulties include the fact that the laser beam is controlled manually from the outside the patient’s body, from a comparatively large range from the surgical site (typically 400 mm). This results in a stringent requirement for direct line-of-sight for laser control, imposing limits on the types of patients that can benefit from this state of the art treatment due to their specific anatomy (Peretti et al., 2016). Furthermore, the long operating range causes laser aiming accuracy and consistency problems, increasing the need for extensive surgical training.

Considering this context, the European project μRALP (executed between 2012 and 2015) pursued pioneering efforts towards a complete redesign of the TOLMS setup and the development of a new flexible endoscopic system for robot-assisted laser phonomicrosurgery (Tavernier et al., 2017). The result was the creation of an advanced micro-surgical robotic system through research on novel robotic endoscopes and precision micro-robotic end effectors, which allowed relocating the imaging sensors and the laser actuator closer to the surgical field. In addition, research in real-time cancer imaging, surgeon-robot interfaces, cognitive controllers, augmented-reality and assistive teleoperation contributed to improve the surgical site visualization, the controllability of the surgical tools, and the accuracy of the operations.

The engineering advancements and scientific contributions of μRALP are reviewed in this paper, together with developments and results achieved beyond and independently of this project. This leads to an outline of the current state of the art and open challenges in the area of robot-assisted endoscopic laser microsurgery.

## Clinical Context and Key Endoscopic Laser Microsurgery Objectives

**FIGURE 1:**
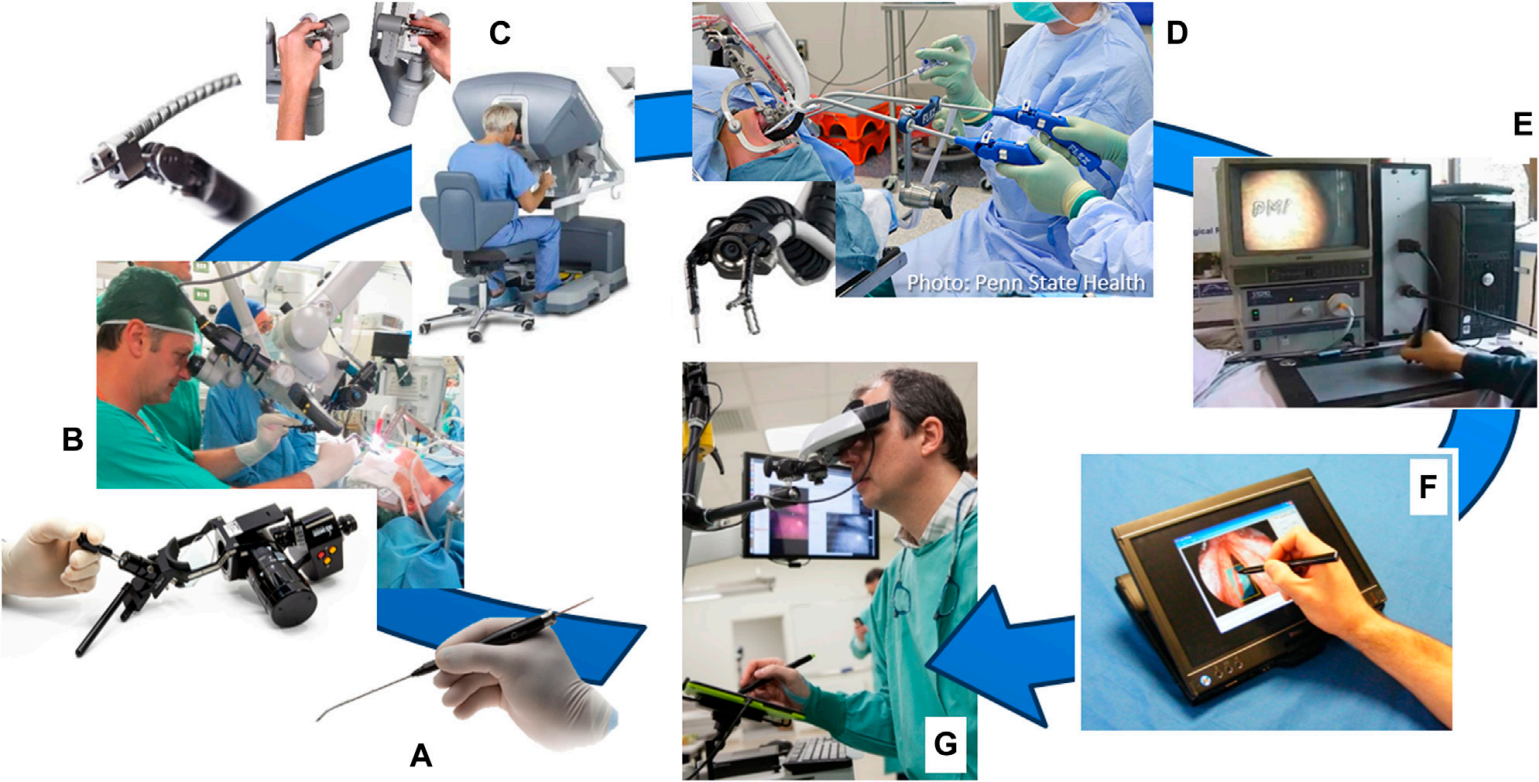
User interfaces for laser surgery. **(A)** Elevate ENT handpiece, an optical scalpel commercialized by OmniGuide Surgical. **(B)** EasySpot Hybrid, a manual laser micromanipulator by DEKA for surgical microscopes. **(C)** Intuitive Surgical’s da Vinci system and **(D)** Medrobotics Flex robotic system, both of which can include an optical fiber for laser surgeries. **(E)** K.U. Leuven’s interface for robot-aided laser surgery based on a graphics tablet (Tang et al., 2006). **(F)** IIT’s Virtual Scalpel interface based on a tablet PC (Mattos et al., 2011). **(G)** µRALP’s Virtual Microscope and tablet-based laser controller (Deshpande et al., 2014).

Back in 2011, a number of clinical devices were already available for laser surgery, including optical scalpels and manual laser micromanipulators commercialized by Deka, KLS Martin, Lumenis, OmniGuide and other companies (see Figure 1). Now, a decade later, emerging robotic technologies still have not replaced the commercial devices for TOLMS. The control of such devices still relies completely on the dexterity and skills of the surgeon, who has to go through a long training process to acquired the expertise needed for phonomicrosurgery. Furthermore, ergonomic issues such as sub-optimal surgeon hand support and the need to operate while looking through a microscope, lower the accuracy and aggravate consistency problems that affect these delicate surgeries.

Nonetheless, the recognition that interfaces (and human factors) play a major role in the success and quality of laser surgeries has driven research into augmenting the surgeons’ capabilities with new surgical systems such as teleoperated robotic devices. In addition, the creation of hollow core optical fibers capable of transmitting CO_2_ laser power has enabled, for example, research into the use of surgical robots, such as the *da Vinci* system (Intuitive Surgical Inc, United States), for laryngeal laser procedures. This possibility was first explored by Solares and Strome (2007), and later by Desai et al. (2008), who have coupled such optical fibers to the *da Vinci*’s tool tip and used it for laryngeal surgeries. This idea was successfully demonstrated by both groups, and later corroborated by others using also other robotic systems, such as the Flex robot launched by Medrobotics Corporation (United States) in 2014 (Lang et al., 2017). In addition, also a more cost effective alternative to robotics based on manual flexible instrumentation has been investigated for laryngeal surgery (Schild et al., 2021). However, the conclusions of such studies continue to emphasize the need for new robotic technologies to improve access, laser aiming precision, and ablation quality for delicate operations in the glottic region. Current robotic instruments are still too bulky for delicate procedures on the vocal cords, limiting their effective use to the oral cavity, pharynx and supraglottic regions. Nevertheless, research continues to push this boundary with the development of small-sized robotic instruments, such as those based on concentric tube technology (Friedrich et al., 2018).

By the time the µRALP project started, research towards new robot-assisted laser surgery systems for minimally invasive soft tissue surgery included the work of Tang et al. (2006) at K.U. Leuven, and Mattos et al. (2011) at IIT. Their research resulted in the creation of writing-based interfaces for controlling laser aiming in robot-assisted surgeries, which demonstrated potential for bringing greatly enhanced precision, controllability, safety, and ergonomics to the operations. However, similarly to the traditional laser microsurgery setups, both systems were still limited by the need for direct line-of-sight from the outside of the patient to the operative field.

Therefore, µRALP and other related research projects have focused on advancing such state of the art in laser phonomicrosurgeries, specially through the elimination of limitations regarding the access to the surgical site and the need for establishing an operative direct line-of-sight from the outside of the patient’s body. For this, the specific concept of µRALP included the creation of a novel teleoperated surgical system based on a micro-robot laser micromanipulator and a custom flexible endoscope, which could bring novel imaging and surgical technologies close to the surgical field. Furthermore, to augment the surgeons’ capabilities, the project also aimed at creating a novel ergonomic and information-rich surgeon-machine interface, including augmented visualization, intuitive controllers and assistive cognitive systems. The ultimate goal was to bringing unprecedented levels of accessibility and precision to laser microsurgeries to allow operations not previously possible with existing technology.

**FIGURE 2:**
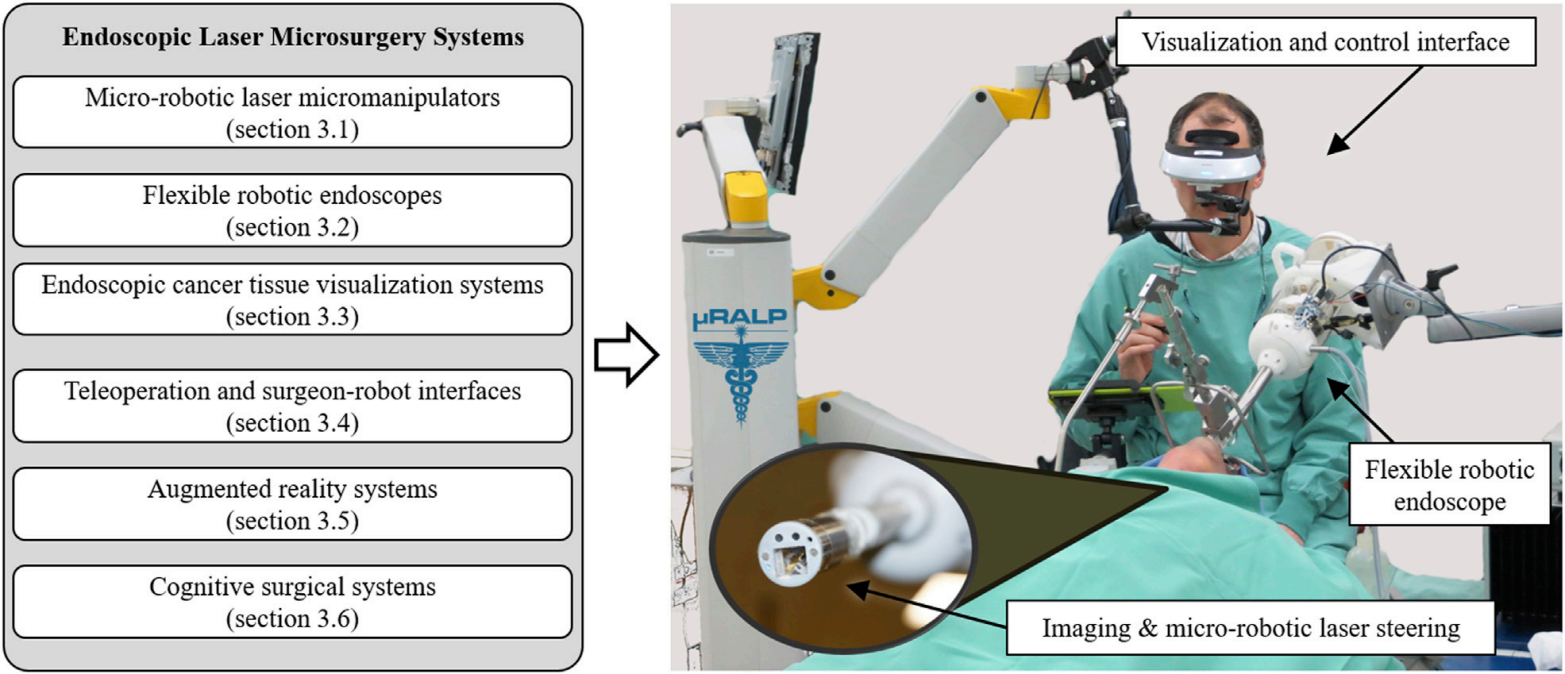
Core technologies of endoscopic laser microsurgery systems reviewed in this paper. These technologies can be integrated to create advanced robotic systems for delicate and high precision operations, such as demonstrated by the µRALP system for robot-assisted laser phonomicrosurgery shown on the right (Tavernier et al., 2017).

To realize this concept, research towards endoscopic laser microsurgery within and beyond µRALP has focused on accomplishing the objectives listed below. These are related to core technologies that can be integrated to create an effective robotic system for endoscopic laser microsurgery, as illustrated in Figure 2. They include:

 • *Micro-robotic laser micromanipulator*: The engineering of a dexterous micro-robotic end-effector for precise high-power laser delivery in minimally invasive surgeries. This system should control the surgical laser steering from the immediate vicinity of the surgical field.
• *Flexible robotic endoscope*: The development of an endoscopic system providing the appropriate degrees of freedom for effective access and visualization of all anatomical subsites of interest.
• *Cancer tissue visualization*: The study and development of micro-optomechatronic technologies and computer vision methods for intraoperative real-time cancer tissue visualization, to support the intraoperative definition of surgical margins.
• *Teleoperation and surgeon-robot interface*: The creation of an intuitive and information-rich augmented reality man-machine interface for assisted teleoperation of the robotic system, including real-time surgical guidance based on pre- and intraoperative surgical plans. This goal involves the design of:
   o An assistive teleoperation interface able to achieve the required control system performances and support informed decisions by the surgeon
   o A laser visual servoing system to guarantee accurate laser steering control and the ability to follow predefined paths, enabling partial automation of surgical tasks
   o An augmented reality surgical interface for accurate preoperative image registration
• *Cognitive surgical system*: The creation of a cognitive system capable of learning and predicting the changing characteristics of the surgical site during laser procedures, to improve laser-tissue interaction quality and safety.

Currently, these objectives continue to be pursued through parallel research and technological development efforts within the research community. Their current state of the art, based on results achieved both within and beyond µRALP, are discussed in the next section.

## Micro-technologies and Systems for Robot-Assisted Endoscopic Laser Microsurgery

The research towards clinically feasible robotic systems for laser microsurgery includes the design, development, assessment and integration of core subsystems. In the case of the µRALP demonstrator shown in Figure 2, such subsystems focused on achieving the objectives outlined above, i.e., the creation of: 1) a micro-robotic system to steer the laser beam; 2) a flexible robotic endoscope to bring the imaging sensors and surgical instruments close to the surgical target; 3) optical technologies and computer vision methods for real-time cancer tissue imaging; 4) teleoperation and surgeon-robot interfaces; 5) augmented reality for enhanced surgical awareness and control; and 6) cognitive systems for safety supervision and autonomous operations. The contributions of µRALP and of research beyond it regarding each of these subsystems are discussed in the next sections.

### Micro-Robotic Laser Micromanipulators

The goal of such micro-robotic devices is to serve as the end-effector of endoscopic laser microsurgery systems, allowing precise laser steering from the vicinity of the surgical field and providing high resolution motions with fast response times. The micro-robot should also allow teleoperation and automatic control schemes (e.g., based on visual servoing methods) to enable high-accuracy operations. For µRALP, the design specifications for the micro-robot included the robot size (diameter ≤10 mm), mobility (laser deflection range ≥30° in two degrees of freedom) and laser aiming accuracy (≤100 µm).

**FIGURE 3:**
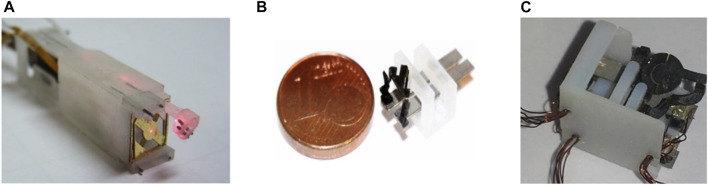
Micro-robotic laser micromanipulators developed during µRALP. **(A)** Squipabot (Rabenorosoa et al., 2014). **(B)** PIBOT (Lescano, 2015). **(C)** Micro Agile-Eye (Lescano, 2015).

During µRALP, three solutions were proposed for the micro-robotic laser micromanipulator. These are presented in Figure 3 and included a hybrid piezoelectric compliant mechanism (Rabenorosoa et al., 2014), and two different piezoelectric smart composite microstructures (Lescano, 2015).

The PiBot and the Micro Agile-Eye piezoelectric smart composite microstructure robots were proposed to satisfy the stringent system requirements by combining the following principles:

• The use of piezoelectric cantilevers allows the achievement of very high positioning resolution (submicrometric).
• The use of several piezoelectric actuators and the lever principle can amplify displacements.
• A parallel kinematic structure allows high miniaturization of the structure while maintaining the range of displacements and of velocities offered by the piezoelectric actuators.
• The use of a 5R (for micro Agile-Eye) and 2 RUS (for the PiBot) parallel kinematic structures allows transforming linear displacements into angular motions for laser scanning with conservation of the high velocity capability.
• The use of smart composites microstructures (SCM) fabrication process can allow microfabrication of the whole piezoelectric microrobot with minimized complexity. The principle consists in machining first the structure in planar form, then folding this in order to obtain the 3D structure.

The Squipabot was the micromechatronic laser micromanipulator finally integrated in the µRALP endoscope. This device was selected for its simple fabrication and assembly methods, and for its higher technology readiness level (TRL) for integration with the other µRALP systems. The Squipabot is based on the use of conventional mechanisms and MEMS technology. More precisely, it is a combination of a compliant micro-fabricated silicon structure (deformable mirror) with innovative commercial linear micromotors (Squiggle motors, NewScaleTech, Victor, NY), which are used to actuate the two decoupled and high range (up to 45°) tilting stages with high accuracy (Renevier et al., 2017).

The Squipabot featured integrated high-resolution magnetic position sensors to determine, in real-time, the position of the linear stages and, consequently, the position of the beam deflection micro-mirror. All components (linear micromotors, MEMS mirror, sensors, laser fiber, fixed mirror, electrical wires) were assembled and packaged in a 3D printed housing. The entire integrated micro-robot, depicted in Figure 3A, measured 9 mm x 11 mm x 42 mm. It successfully satisfied the performance requirements by demonstrating closed-loop trajectory following root-mean-square (RMS) errors in the order of 80 μm, laser deflection velocity up to 95°/s, and control loop frequency up to 40 Hz. Furthermore, the laser steering system demonstrated the same level of performance during cadaver trials, even though the controller frequency was decreased to 17 Hz due to limitations in the RGB cameras frame rate.

#### Micro-Robotic Laser Micromanipulators Beyond µRALP

About the same time the µRALP project started, other research groups were also investigating technologies for endoscopic laser microsurgery. Hoy et al. (2011) proposed a 9.6 mm diameter femtosecond laser microsurgery probe based on a 2-DoF MEMS-scanning mirror. The system presented resonant optical deflections of ±7.1° and ±15.3° at 2.26 and 0.98 kHz. The same research group later miniaturized the probe design based on the use of a 2-DoF piezoelectric fiber scanner (Ferhanoglu et al., 2014), which resulted in a 5 mm diameter probe with resonance frequencies around 900 Hz and scan area of 150 μm × 150 μm. In addition, Patel et al. (2012) proposed an endoscopic laser scalpel able to steer a laser beam using two optical wedges controlled by miniature piezoelectric motion systems, achieving a device that was 5 cm long by 17 mm diameter.

**FIGURE 4:**
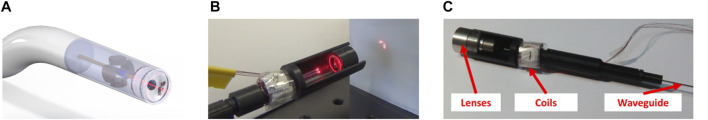
Magnetically actuated laser scanners for endoscopic microsurgery. **(A)** Concept **(B)** Prototype based on a standard silicon optical fiber (Acemoglu et al., 2019). **(C)** Prototype based on a CO_2_ laser waveguide (Acemoglu and Mattos, 2018).

Research beyond the end of µRALP continued to pursue higher TRL and alternative technological solutions for micro-robotic laser micromanipulation. These efforts resulted, for example, in the creation of a magnetically actuated laser scanner for endoscopic microsurgery (Acemoglu et al., 2019), which demonstrated a 133 mrad × 133 mrad scan range and open-loop accuracy below 1.4 mrad for scanning frequencies up to 15 Hz. These results translated into a 4 mm × 4 mm range with 90 μm accuracy at 30 mm working distance. The device, depicted in Figure 4, is based on the creation of a local magnetic field to bend a cantilevered laser fiber in a controllable fashion. It was originally based on the use of a standard silicon optical fiber with 300 µm core diameter, but was subsequently enhanced to use a waveguide for CO_2_ lasers. This allowed demonstrating higher quality tissue ablations when compared to the bare waveguides currently in clinical use, enabling both reduced carbonization levels and narrower ablation craters (Acemoglu and Mattos, 2018). The concept of this device was later extended to allow closed-loop control of the scanning fiber, demonstrating promising results towards a system with higher accuracy (Mohammadbagherpoor et al., 2019).

**FIGURE 5:**
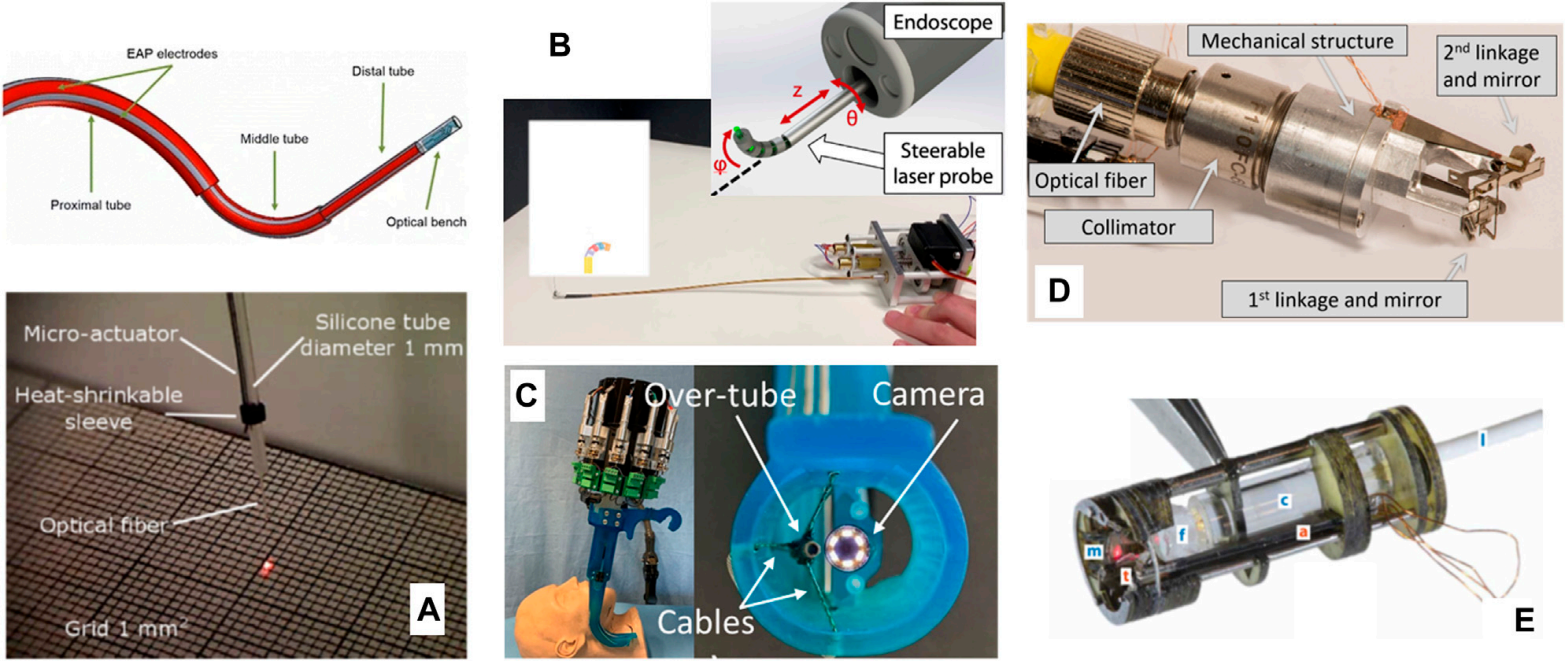
Further laser steering devices for endoscopic surgery. **(A)** A biocompatible conducting polymer continuum robot (Chikhaoui et al., 2018) **(B)** A flexible steerable instrument (O’Brien et al., 2019). **(C)** A cable-driven parallel robotic system (Zhao et al., 2020). **(D)** A millimeter-scale tip/tilt laser-steering system (Bothner et al., 2019). **(E)** Microrobotic laser steering system (York et al., 2021).

Other prototypes proposed beyond µRALP to steer laser fibers for microsurgery include biocompatible conducting polymer continuum robots (Chikhaoui et al., 2018), tiny flexible steerable instruments to be used through the tool channel of clinical endoscopes (O’Brien et al., 2019), and a cable-driven parallel robotic system for phonosurgery (Zhao et al., 2020). Finally, a millimeter-scale tip/tilt laser scanning system based on a micro-mechatronic structure actuated by piezoelectric beams has been proposed for transoral robotic surgery, demonstrating a field of view of 10 mm x 20 mm and scanning speed up to 7 m/s from an 11 mm diameter device (Bothner et al., 2019). This device was subsequently further miniaturized to a diameter of 6 mm and improved to cover an 18 mm x 18 mm workspace (York et al., 2021). Images of such systems are presented in Figure 5. Table 1 and a recent review by Fichera (2021) further summarizes emerging technologies for laser deflection within the body.

**TABLE 1:** State of the art robotic laser manipulators for endoscopic laser microsurgery.

Reference	Size (mm)	Length (mm)	Laser steering technology	Max. optical beam deflection (degree)	Working distance, WD (mm)	Resonant frequency of each axis (Hz)	Maximum speed at WD (mm/s)	Range of motion at WD (mm)	Laser type^a^	Illumination and imaging systems^b^
Hoy et al. (2011)	9.6 ⌀	23	MEMS mirror	14.2 x 30.6	≈0.5^d^	2260, 980	641^d^	0.142 x 0.297	Femtosecond	Included (same system for imaging and ablation)
Patel et al. (2012)	17 ⌀	50	Wedge lenses	56 ⌀^d^	30^c^	―	127^c^	31.8 ⌀^c^	CO_2_	Included, 2D
Ferhanoglu et al. (2014)	5 ⌀	40	Piezoelectric fiber scanner	4.3 ⌀^c^	2	895, 904	270^d^	0.15 x 0.15	Femtosecond	Included (same system for imaging and ablation)
Renevier et al. (2017)	11 x 9	42	MEMS mirror	45 x 45	20	―	33^d^	20 x 20	Er:YAG	No
Acemoglu and Mattos (2018)	13 ⌀	60	Electromagnetic fiber scanner	7.6 ⌀^d^	30	63, 63	504^d^	4 ⌀	CO_2_ and 1470ƞm diode laser	No
Chikhaoui et al. (2018)	2 ⌀ ^e^	30^e^	PPy-based fiber scanner	10 ⌀	57^d^	―	0.166^d^	5 ⌀	Low power visible laser	No
Kundrat et al. (2019)	11.5 ⌀	70^e^	Continuum robot	97 ⌀^d^	20	―	3.5	45 x 45	Er:YAG	Included, 3D
Zhao et al. (2020)	22 x 20	―	Cable-driven parallel robot	30 ⌀	1	―	3	14 x 28^e^	2 μm thulium laser	Included, 2D
York et al. (2021)	6 ⌀	16	Mirrors actuated by piezoelectric beams	40 x 40^d^	25	1800, 1900	3900	18 x 18	Low power visible laser	No

a Surgical laser type use during system assessment based on published data.

b Illumination and imaging systems included in the listed dimensions.

c Calculated based on published data assuming WD = 30 mm as a suitable distance for endoscopic laser microsurgery.

d Calculated from published data.

e Estimation based on published images and data.

### Flexible Robotic Endoscopes

The specific objective of this device in the context of endoscopic laser microsurgery is to provide a robotic structure to deploy, support, position and properly orient the imaging and laser steering systems to allow effective operations. This requires the creation of a device with the appropriate size, operative channels and degrees of freedom to access the anatomical sites of interest (e.g., the larynx).

In µRALP, the development of such endoscopic system was an iterative process strongly influenced by results of adjunct research and cadaver trials. The final device consisted of the following components: a distal tip (housing a stereo imaging system, illumination fibers, the Squipabot, and laser focusing optics), one bendable and extendable continuum segment, one solely bendable continuum segment, a rigid shaft and an actuation unit (Kundrat et al., 2015).

The endoscope’s actuation unit provided manual and motorized actuation for the two consecutively attached continuum segments. Actuation was based on spindle driven carriers, which were attached to NiTi rods and wires connected to both segments and guided through the rigid shaft. Each segment was actuated by three rods and wires. Manual actuation was connected to the first segment and allowed for in-plane bending (1 degree of freedom, DOF). Intraoperative positioning of the Squipabot was achieved with the second continuum segment. The flexible and leak-tight continuum segments were manufactured individually by silicone casting. The flexible segments were rigidly connected to the distal tip and rigid shaft. Three motors actuated the spindle-carrier system, enabling bending in two DoF (pan-tilt) and extension of the segment. Another DOF was achieved by manually rotating the actuation unit inside the customized interface.

Control of the endoscope’s actuation system was implemented on a BeagleBone Black embedded Linux device. A customized extension was designed to connect the motors directly to the RS232 interface and power supply. Customized Robot Operating System (ROS) modules provided low and high level interfacing with the µRALP control framework. Finally, the kinematics of the actuated continuum segment was derived to enable automatic control (Kundrat et al., 2015).

The endoscope distal tip provided central alignment for the Squipabot (see *Micro-robotic laser micromanipulators*). In order to obtain an overlapping workspace, the imaging sensors and illumination light guides were circumferentially aligned and inclined with respect to the laser steering micro-robot. Optical fibers and electrical cables were routed within the endoscope in order to be protected during intraoperative handling.

The robotic endoscope design also considered different approaches for stabilizing the system with respect to the patient. The decision to use a commercial manually lockable positioning arm was taken after preliminary cadaver experiments, since it demonstrated proper support while being readily available. This support consisted of two parts: a serial kinematics arm and a custom interface to the endoscope unit. The custom interface added two additional DOFs to the supporting system, facilitating intraoperative handling. In addition, access to the laryngeal anatomy was facilitated by the use of a commercial mouth retraction device, allowing improved dexterity for inserting the µRALP endoscope through mouth and oropharynx to finally reach the laryngopharynx.

#### Flexible Robotic Endoscopes Beyond µRALP

**FIGURE 6:**
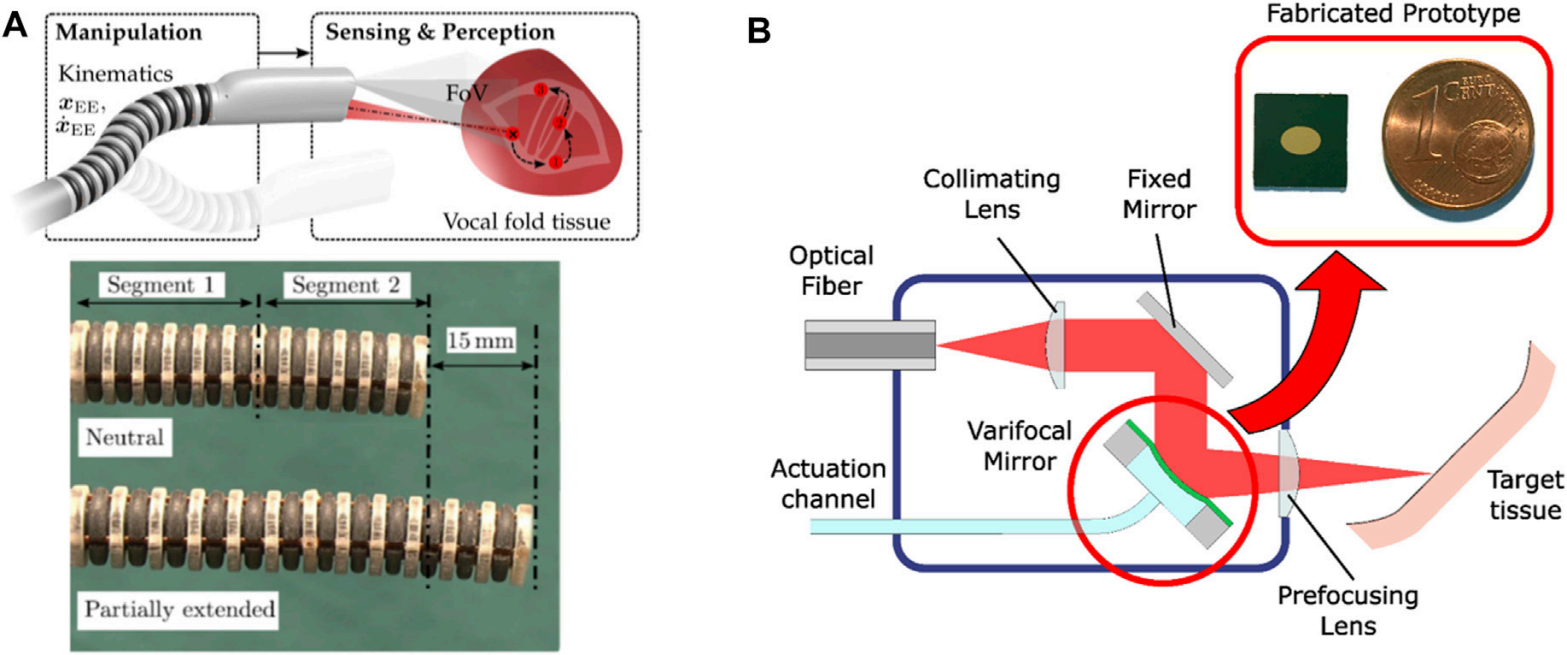
**(A)** Extensible hollow core continuum robot for non-contact laser surgery (Kundrat et al., 2019). **(B)** High power laser auto-focusing system based on a hydraulically actuated MEMS varifocal mirror (Geraldes et al., 2019).

The realization of robotic endoscopes for delicate laser microsurgeries has continued to be pursued beyond the µRALP project. This included efforts towards systems with higher TRL, such as work towards the integration of high power surgical lasers into the endoscopes, and the design of novel mechanisms for higher robustness and performance. Non-contact soft tissue ablation was demonstrated using a Er:YAG laser (2.94 µm wavelength), delivered using a GeO_2_ solid core fiber and appropriate laser focusing optics (Kundrat et al., 2016). Subsequently, a new five DOFs continuum robotic endoscope composed of two extensible segments with 11 mm outer diameter and a large inner lumen of 5.75 mm was designed and fabricated monolithically. The system, depicted in Figure 6A, featured multiple rigid guidance elements connected to bellow-shaped flexible sections to enable bending, extension, and compression of the structure, which demonstrated bending up to 90° and elongation of up to 80% from its initial length. These capabilities were instrumental to allow the demonstration of assistive and autonomous technologies for laser focus adjustments (Kundrat et al., 2019), which were subsequently assessed through phantom studies with clinical experts (Kundrat et al., 2020).

The realization that high power laser focusing is critical for the precision and quality of endoscopic laser microsurgeries also lead to parallel research into micro-opto-electromechanical systems (MOEMS) for this purpose. This included the development of a dynamic focusing system based on a very small (3 × 4.24 mm^2^) hydraulically-actuated MEMS varifocal mirror, which was able to adjust the focal length of the laser without moving the tip of the endoscope. The concept and an image of the micro-fabricated mirror are shown in Figure 6B. This device proved to be appropriate for use with high power surgical lasers (including CO_2_ lasers) and enabled the implementation of an auto-focusing system with focal length ranging from 12 to 52 mm (Geraldes et al., 2019).

### Endoscopic Cancer Tissue Visualization Systems

The goal of such systems in the context of high-precision endoscopic microsurgery is to support the detection of tumors and the intraoperative definition of surgical margins. During µRALP, this was pursued through research and development of optical technologies and computer vision methods for enhanced real-time visualization of cancer tissue, which led to the development of a dual imaging system for the acquisition of stereoscopic white-light and fluorescence images.

The white-light imaging system was specifically designed for high-speed imaging to enable visual servoing of the laser beam steered by the Squipabot. It was based on the use of two imaging bundles of 50,000 fibers each and a high-speed camera, providing monochrome stereo images at up to 1,000 frames per second (fps). The system’s field of view was 15 mm in diameter at a 25 mm working distance, resulting in a pixel resolution of approximately 13 µm/pixel.

The fluorescence imaging system was based on the same fiber bundles and an additional fluorescence excitation laser. Optical filters were used outside the endoscope body to select the wavelengths of interest. The system was able acquire 10 fluorescence images per second (10 Hz), which were automatically co-registered and with the same pixels resolution as the white-light images.

**FIGURE 7:**
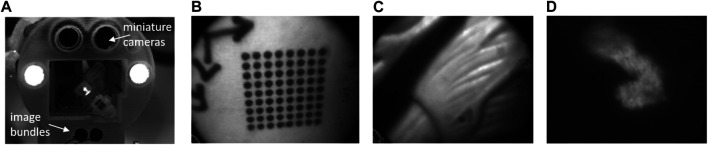
**(A)** the µRALP endoscope tip with integrated dual imaging system (Tavernier et al., 2017). **(B)** and **(C)** white-light images acquired at 600 fps. **(D)** Fluorescence image acquired with the same imaging bundle.

The realization of this dual imaging system demonstrated a new hardware for hyperspectral augmented-reality visualization of the surgical field. Figure 7 shows a picture of this system integrated to the µRALP endoscope tip and sample images acquired with it at 600 fps, which presented satisfactory resolution, contrast and field of view.

In addition to the development of new hardware for cancer imaging, µRALP also involved research on computer vision methods for automatic detection and classification of laryngeal tumors based on narrow-band imaging (NBI) endoscopic videos. The primary goal here was to help surgeons better delineate tumor margins, to then precisely excise them with the surgical laser. Nonetheless, such computer vision methods are expected go much further and enable the optical biopsy of lesions. Research in this direction started around 2011, when clinical studies were starting to establish correlations between the characteristics of laryngeal mucosal microvascular network and different cancer types (Ni et al., 2011). Therefore, the automatic recognition of microvascular patterns was deemed as a promising technology to assist in cancer detection and surgical margins definition.

Initial research on this topic focused on detecting and classifying blood vessel patterns based on anisotropic filtering, morphological analysis, and statistical analysis of extracted metrics such as blood vessels’ thickness, tortuosity, and density (Barbalata and Mattos, 2016). The method reached an overall classification accuracy of 84.3% during a preliminary assessment, proving the feasibility of the approach.

#### Endoscopic Cancer Tissue Visualization Systems Beyond µRALP

Following up on the promising results achieved during µRALP, further research by project partners included the development of AI-based endoscopic image analysis methods for laryngeal tissue classification. Initially, this was pursued through texture analysis of NBI image patches (Moccia et al., 2017), which achieved a median recall of 98% on a well-balanced dataset built from endoscopic videos of 33 patients. Subsequently, with the development of Deep Learning methods, the work progressed to the automatic analysis, segmentation and classification of tumors in full endoscopic video frames (Paderno et al., 2021a).

The use of computer vision and AI to enhance cancer tissue detection and diagnosis on endoscopic videos is now a large research topic. Particularly, it includes research and development of assistive systems termed CADe (Computer-Aided Detection) and CADx (Computer-Aided Diagnosis). Progress in this area has been strongly linked to the gastro-intestinal (GI) endoscopy field (Sumiyama et al., 2021). For this area, clinical CADe systems such as the Olympus’ ENDO-AID CADe^1^ and Medtronic’s GI Genius^2^ are already available. However, the development of CADx systems, which are expected to improve early lesion detection, classification and characterization, is still restricted to research (Paderno et al., 2021b).

In the ENT field, the development of AI-based CADe and CADx systems are also progressing fast. This is being accelerated specially by large efforts in building extensive databases of endoscopic videos, annotated images and associated clinical data, which are required to train the AI systems. Based on such large datasets, CADe systems are already demonstrating remarkable overall accuracy of 96% for the detection and classification of laryngeal diseases such as nodules, polyps, leukoplakia and cancer (Ren et al., 2020).

### Teleoperation and Surgeon-Robot Interfaces

In the context of endoscopic laser microsurgery, the specific goals of teleoperation systems include the development of hardware and software infrastructure for the remote operation of the surgical endoscope, and the implementation of appropriate user interfaces for intuitive and precise control of the system by the surgeon.

Initial efforts in this direction focused on comparing different types of laser control interfaces in terms of usability, precision and ergonomics (Dagnino et al., 2011). Results from this study corroborated the selection of a stylus and tablet as the most intuitive and precise interface for the task. Consequently, subsequent research focused on a comprehensive assessment of the Virtual Scalpel system involving expert and novice ENT surgeons (Mattos et al., 2014). This system, shown in Figure 1F, allowed the use of a stylus to control the steering and activation of the surgical laser beam directly from a touch-screen monitor, where real-time video of the surgical site was displayed. Results demonstrated the Virtual Scalpel could augment the surgeons’ skills by providing a highly intuitive control interface able to eliminate the hand-eye-foot coordination issues that affect the standard laser microsurgery systems used clinically. This translated into significantly enhanced laser aiming accuracy and controllability, as assessed through a quantitative analysis of trajectory following errors.

**FIGURE 8:**
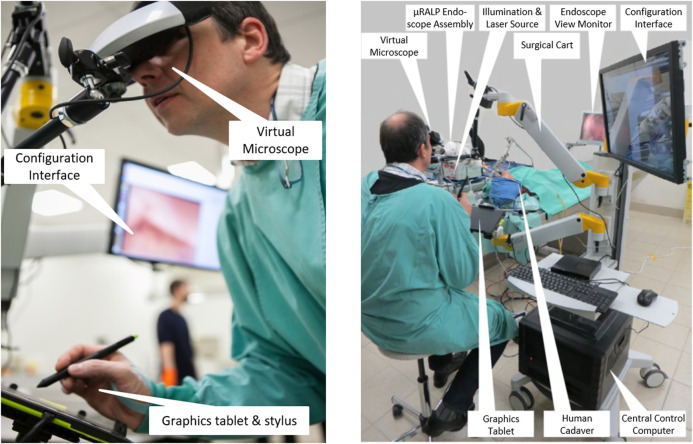
The µRALP teleoperation interface and its components (Deshpande et al., 2014).

However, feedback from the surgeons also highlighted the need for stereoscopic visualization of the surgical site for proper depth perception during the delicate laser microsurgeries. Therefore, the Virtual Scalpel system was redesigned to provide such visualization. This lead to the development of the Virtual Microscope concept (Deshpande et al., 2014), in which a stereoscopic head-mounted display (HMD) was used to simulate a standard surgical microscope, and a graphics tablet was used as the input device for controlling the laser beam. Results here demonstrated similar performance enhancements as Virtual Scalpel system in terms of laser control accuracy and usability, with the extra benefits of allowing 3D visualization and augmented reality features. Therefore, this was the surgeon interface selected for the final µRALP system (Figure 8).

Overall, the final µRALP surgeon interface was composed of the following main elements:

 • Input Interface: A graphics tablet was used for laser aiming control. Buttons on the stylus were used for the definition of intraoperative plans and for system configuration changes.
• Visualization Interface: The system included three visualization devices:
   o Virtual Microscope: This component provided real-time stereoscopic visualization of the video streams produced by the endoscope’s imaging system. The 3D videos were displayed on a high-definition immersive stereoscopic display fixed to the µRALP surgical cart with an adjustable arm.
   o Configuration Interface: A touchscreen monitor was used for system configuration, operating mode selection, alarm messages, and as a supplementary display for surgical site visualization.
   o Endoscopic View Monitor: An additional monitor was used to display the real-time endoscopic video for the surgical team in the operating room. It also served as a visualization aid during the manual insertion and rough positioning of the µRALP endoscope near the surgical site.
• Surgical Cart: A cart was used to integrate and organize the different parts of the surgical system into a single rack-style configuration. It provided housing and support for the system’s control computer, graphics tablet, Virtual Microscope, and configuration touchscreen monitor. It was designed to be easily rolled in and out of operating rooms and reconfigurable to match the surgeon requirements, allowing adjustable positioning of the interface devices from a small footprint system.

Controlling the complete µRALP surgical system from the surgeon interface required full software and hardware integration and real-time operations. A custom system architecture was implemented for this based on the Robot Operating System (ROS), as described by Deshpande et al (2014). The software components included: Input command processing; Image acquisition, processing, and display; Visual servoing for closed-loop laser control; Image registration and 3D reconstruction; Augmented reality processing and display. The hardware components included: Micro-robotic laser micromanipulator (Squipabot); Robotic endoscope; Illumination; Endoscopic cameras; Visualization devices.

In addition, µRALP’s integration and control system included both software and hardware components to ensure operational safety. This included the use of an embedded real-time safety watchdog, hardware interlocks and a conventional footswitch, all of which could deactivate the surgical laser and the robotic endoscope in case of errors or when commanded by the system’s software.

**FIGURE 9:**
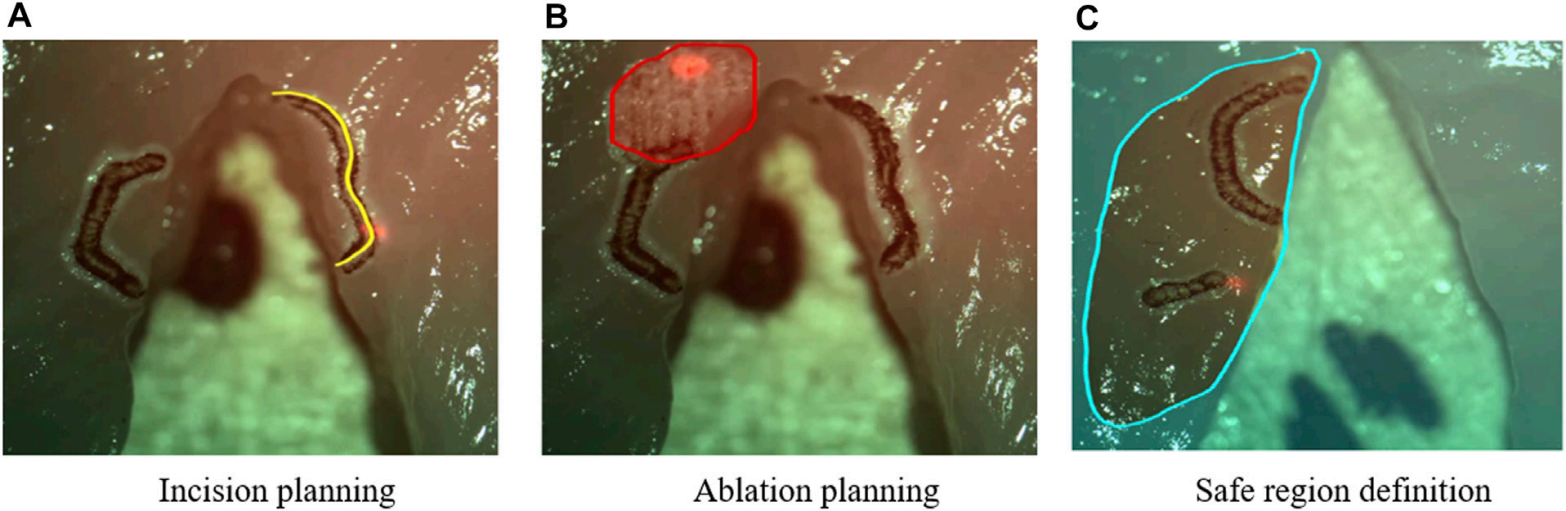
Examples of intra-operative planning of incisions paths, ablation patterns, and safety regions based on graphic overlays (Deshpande et al., 2014). The high-power surgical laser was only enabled within the defined safe region.

When using the µRALP system, the surgeon was in full control of the operation. Nonetheless, different components assisted in the execution of surgical tasks. These provided the following assistive features:

1) Virtual Scalpel: Real-time laser aiming control using the stylus and tablet interface.
2) Intraoperative Planning: The stylus could be used to define scan patterns in the surgical field, allowing the planning of incisions or ablation regions for subsequent automatic execution (Figure 9).
3) Predictive Safety: The stylus could also define safe and forbidden virtual regions in the surgical field, which were used as virtual fixtures to automatically enable or disable the high-power surgical laser.

Accurate automatic execution of surgeon-defined intraoperative plans was pursued through research on novel laser visual servoing methods. This resulted in the development of two methods, called epipolar and trifocal visual servoing. The epipolar method used one of the embedded cameras and Squipabot’s actuated mirror as a virtual camera to implement a weakly calibrated controller able to accurately follow paths in a 3D scene (Andreff et al., 2013). This method was also shown to enable decoupling path following from velocity profile control, offering advantages in terms of laser-tissue interaction control (Seon et al., 2015).

**FIGURE 10:**
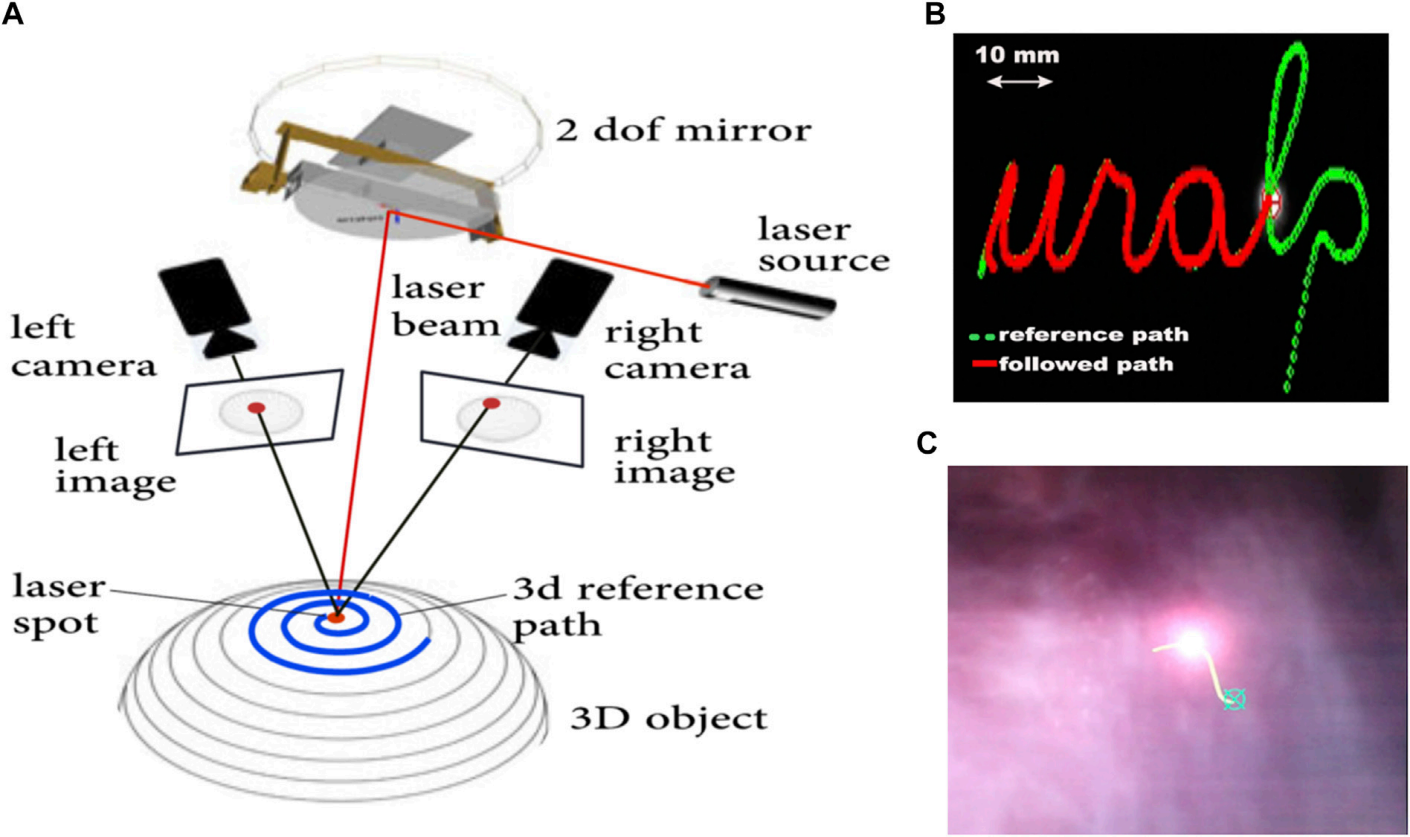
**(A)** Schematic view of a trifocal laser visual servoing system with two cameras (Tamadazte et al., 2018). **(B)** Trajectory following test on a 2D surface (Seon et al., 2015). **(C)** Intraoperative scene during laser visual servoing on the vocal cord of a cadaver (Andreff and Tamadazte, 2016).

An schematic view of the trifocal visual servoing method is presented in Figure 10A. It used the two endoscopic cameras and the actuated mirror (virtual camera) to construct a three-view imaging system and then use the trifocal constraint to design a robust and accurate controller (Andreff and Tamadazte, 2016). This method was shown to simplify the eye-to-hand visual control law of the pan-tilt laser, avoiding the need for a strong Euclidean calibration of the system (camera-robot calibration) and for interaction matrix inversions. At the same time, it provided good performance, achieving an RMS error of 1.20 pixels in trajectory tracking tasks during cadaver trials with the µRALP system.

#### Teleoperation and User Interfaces for Endoscopic Laser Microsurgery Beyond µRALP

Research beyond the end of µRALP continued the efforts towards fast, accurate and robust laser visual servoing, leading to the development of a new path following method incorporating trifocal constraint (Tamadazte et al., 2018). This method ensures accurate 3D control of laser spot displacements in unknown environments while exhibiting good robustness with respect to the calibration and measurement errors and scene variations. In addition, it allows perfectly decoupling the laser spot velocity from the path shape.

**FIGURE 11:**
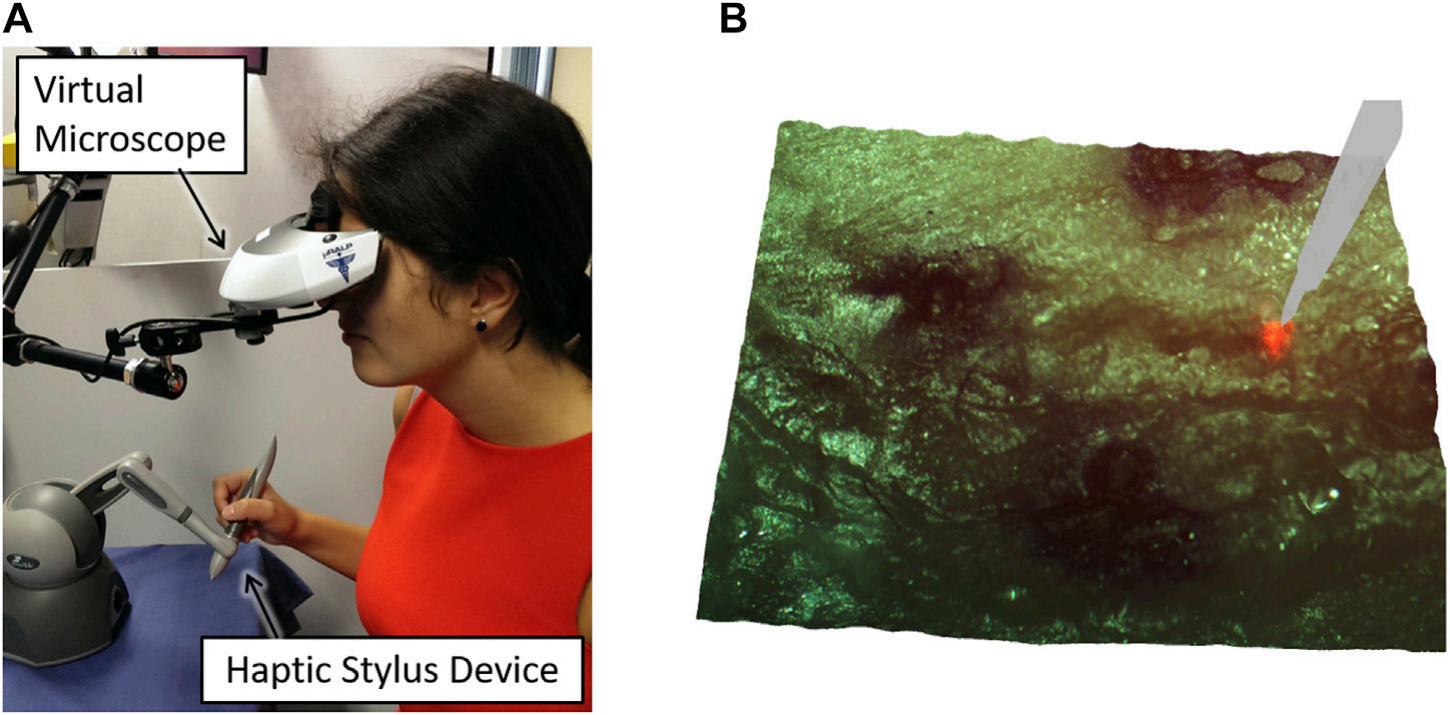
The Haptic Laser Scalpel, developed to bring the sense of haptics to contactless laser surgeries (Olivieri et al., 2018). **(A)** Surgeon interface **(B)** 3D visualization of the surgical site with a virtual haptic scalpel avatar.

Furthermore, continued research towards surgeon interfaces with improved usability, intuitiveness, and laser control performance led to the development of the Haptic Laser Scalpel system (Olivieri et al., 2018). This new control interface, depicted in Figure 11A, brought the sense of haptics to contactless laser surgeries, enriching the surgeon experience and allowing the exploitation of active constraints and guidance techniques to significantly enhance laser control accuracy both in static and dynamic environments. This was realized by exploiting stereoscopic visualization and real-time 3D reconstruction to create a virtual haptic surface representing the real surgical site, which could be explored using a commercial haptic device. This same device was also used to control the steering of the surgical laser beam, allowing the co-location of the haptic feedback and the laser spot seen on the target tissue.

Subsequently, the potential of the Haptic Laser Scalpel as a novel visuo-haptic feedback system was extended and further explored as a new surgical training method. For this, a virtual laser scalpel avatar was added to the surgical scene as shown in Figure 11B, and the system was integrated with a neuro-feedback system that could both monitor the user’s mental focus and enable/disable the activation of the high-power surgical laser. This was shown to assist in training the users to maintain high mental focus during laser control tasks (Barresi et al., 2015).

More recently, different feedback concepts were assessed for precise teleoperation of focal adjustment in endoscopic non-contact laser surgery using an extensible continuum robot (Kundrat et al., 2019). Novice and expert users conducted adjustment tasks and were additionally provided with haptic, (augmented) visual and visuo-haptic feedback. It was demonstrated that the combined feedback involving visual and haptic cues significantly lowered the residual focal errors.

### Augmented Reality Systems for Endoscopic Laser Microsurgery

Augmented reality (AR) in endoscopic microsurgery can be used for enhancing surgical site visualization, to enable intraoperative surgical planning, and to allow the implementation of autonomous controllers based on stereoscopic methods. Within µRALP, surgical AR research included the development of methods for planning laser incisions in 3D, for assessing and controlling the laser focus, and for creating image overlays based on information from the tissue surface and from the laser.

**FIGURE 12:**
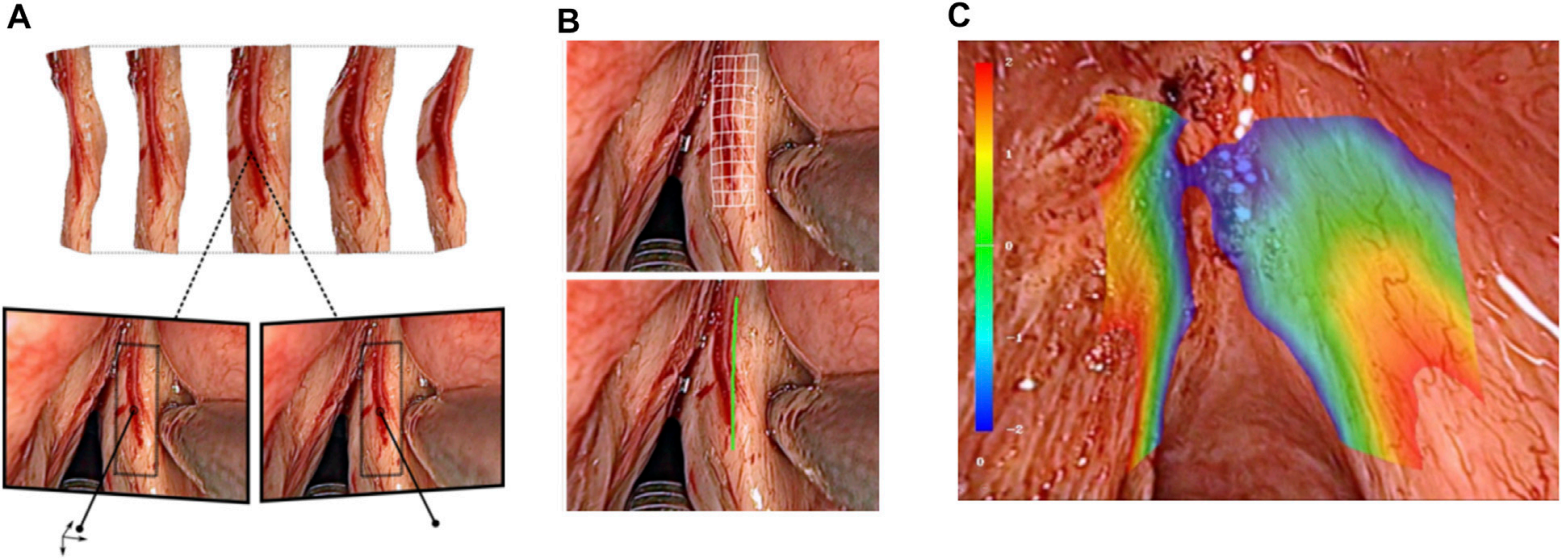
Real-time stereoscopic methods for **(A)** 3D reconstruction (Schoob et al., 2015). **(B)** intraoperative incision planning and visualization (Schoob et al., 2017), and **(C)** laser focus adjustment (Schoob et al., 2016).

One of the main achievements in this area regarded real-time intraoperative acquisition of tissue surface information (see Figure 12). For this, a fast 3D reconstruction method providing sub-pixel accuracy at up to 25 frames per second was developed based on stereo image processing (Schoob et al., 2015). This corresponded to a reconstruction accuracy below 1 mm when using the µRALP endoscope, which featured a stereo imaging system with working distance between 20 and 30 mm. Furthermore, the method included robust techniques for outlier rejection and for handling radiometric illumination changes, as these naturally occur in the tube-like larynx.

Image-based assistance to the surgical workflow was achieved by incorporating the extracted tissue surface information in the definition and visualization of surgical plans. For this, a new method for visual augmentation and three-dimensional feedback was developed. The method included real-time registration of the laser workspace on the live stereoscopic view, enabling accurate registration of laser incision plans with a maximum error of 0.2 mm (Schoob et al., 2016).

Tissue surface information was also used to produce a synthetic laser view, which was exploited in the implementation of assistive and automatic laser focusing methods (Schoob et al., 2015). This included an intuitive framework for interactive laser focus positioning, which used color-coded image overlays to highlight regions in the surgical site under proper laser focusing (Schoob et al., 2016b). The system was shown to allow manual positioning of the laser focal plane on the target tissue with an accuracy of 0.4 mm within seconds.

#### Surgical AR for Endoscopic Laser Microsurgery Beyond µRALP

Research beyond the end of µRALP continued the development and enhancement of these assistive systems for endoscopic laser surgery, introducing extensions able to compensate for tissue motion and tracking inaccuracies such as inconsistent feature matching and drift. The enhanced framework proved to be suitable for online ablation control in laser microsurgery, enabling accurate execution of laser incision paths defined by the surgeon even in the presence of tissue motions and deformations (Schoob et al., 2017). The system demonstrated real-time operation and highly accurate soft tissue tracking performance, providing tracking errors below 0.05 mm and path ablations with RMS error below 0.21 mm in dynamic conditions. Subsequently, this system was integrated into the controller of a new robotic endoscope for non-contact endolaryngeal laser surgery, enabling novel visualization concepts for eye-in-hand laser manipulation, as demonstrated in a user study with novice and expert users (Kundrat et al., 2020).

### Cognitive Surgical Systems for Endoscopic Laser Microsurgery

The development of cognitive systems within µRALP aimed at providing safety supervision and autonomous operations to further improve the safety, quality, and precision of laser microsurgeries. This led to research towards the modeling and control of laser-tissue interactions, which are critically important in delicate tissue sparing operations such as laser phonomicrosurgery. In fact, after the complete eradication of malignancies, a secondary major clinical goal in this case is the preservation of healthy tissue to maintain key larynx functionalities and enable good post-treatment vocal quality.

From a research and technology development perspective, this clinical requirement translates into the need to perform precise and clean laser cuts on the soft laryngeal tissue, avoiding carbonizations and thermal damage to surrounding healthy tissue. In addition, the depth of laser ablations should be properly controlled, to avoid damaging underlying tissue layers. Satisfying these needs requires controlling the laser-tissue interaction process. This was pursued within µRALP not only through laser focus control and laser scanning capabilities as discussed above, but also through the development of cognitive systems to model and control the laser-tissue interactions in real-time. Initially, tissue temperature dynamics under high-power laser irradiation was studied and reliably modeled using nonlinear regression based on Gaussian basis functions (Pardo et al., 2015). This knowledge was then used to generate real-time estimates of the thermal state of soft tissues during laser ablation, proving the approach was suitable to produce feedback for automatic laser incision control (Pardo et al., 2014).

**FIGURE 13:**
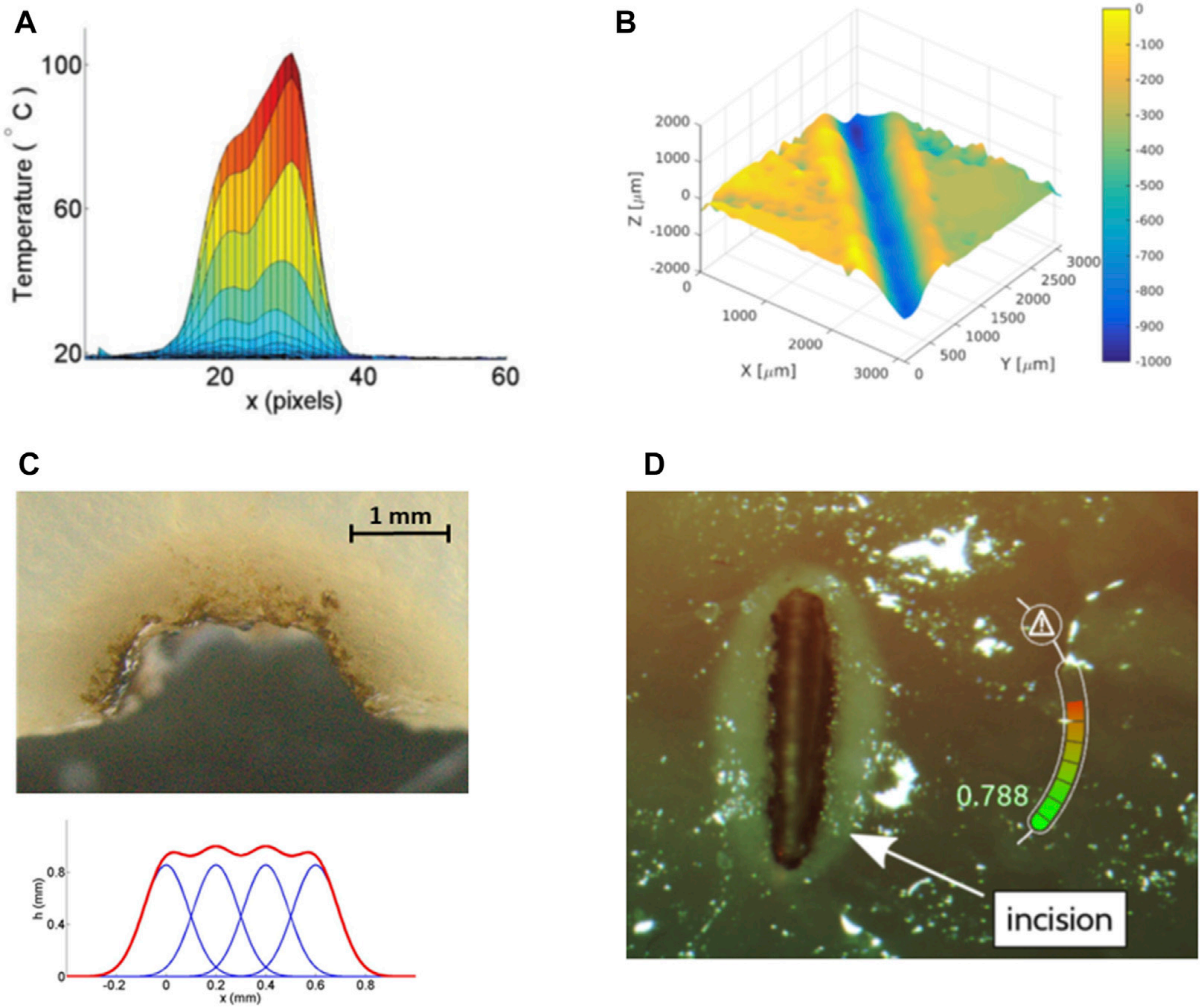
Results from research on cognitive modeling and control of laser-tissue interactions. **(A)** Real-time estimate of superficial tissue temperature during high-power laser scanning (Pardo et al., 2014). **(B)** Autonomous laser incision depth control along incision paths (Acemoglu et al., 2019). **(C)** Autonomous tissue volume vaporization by laser ablation (Fichera et al., 2015b). **(D)** Augmented reality gauge for displaying the laser incision depth progression in real-time (Fichera et al., 2016).

Subsequent research focused on the modeling, online estimation and automatic control of the laser incision depth in soft tissues (see Figure 13). This resulted in the development of a model able to estimate, in real-time, the depth of laser ablations with RMS error of 0.1 mm for depths ranging up to 1.4 mm (Fichera et al., 2015a). This model was then used in a robotic laser microsurgery system to enable both autonomous laser incision depth control along cutting trajectories and autonomous ablation of tissue volumes based on feed-forward control strategies (Fichera et al., 2015b). Finally, these controllers were extended to allow regulating the laser energy density along incision paths, demonstrating that target depths could be achieved within ±60 µm error range (Acemoglu et al., 2017).

The developed laser-tissue interaction models and controllers were also integrated into µRALP interface to provide assistive functions during surgery. For instance, methods to provide real-time feedback on the laser incision depth to the surgeon were researched. These included the use of an augmented reality gauge to display the incision depth progression (Fichera et al., 2015a), and of kinesthetic and vibrotactile haptic feedback to inform the user when a target depth was reached (Fichera et al., 2016). Both systems were shown to significantly enhance the laser incision depth control capabilities of the users during preliminary trials.

#### Cognitive Surgical Systems for Endoscopic Laser Microsurgery Beyond µRALP

In view of developing cognitive systems for future autonomous surgery, research beyond µRALP by project partners included the development of AI-based computer vision systems for the automatic detection of vocal folds, of general anatomical relationships, and of instruments present in the surgical field. For this, an open access dataset with annotated endoscopic images was created, and the performance of different convolutional neural networks were compared. An ensemble network of UNet and ErfNet reached a jaccard similarity index for laryngeal soft tissue of 84.7% (Laves et al., 2019).

More recently, other research groups also started to investigate cognitive systems for soft tissue laser surgery, proposing similar feed-forward automatic controllers for volumetric resections during brain surgery (Ross et al., 2018), and characterizing photoablation crater profiles considering a range of laser incidence angles, which can impact laser ablation models (Ma et al., 2020).

### Integrated Endoscopic Laser Microsurgery System and Cadaver Trials

The realization of a laser microsurgery endoscopic system feasible for clinical use requires the integration of the core technologies described above. This is not a trivial task as it involves both software and physical hardware integrations while respecting all application requirements in terms of size, flexibility, degrees of freedom, workspace, controllability, usability and safety. However, if the technologies are co-developed considering integration goals from the beginning, the integration process can be facilitated and become more efficient. This was the case for the µRALP project, which also involved a series of cadaver trials over 3 years for the testing and validation of integrated system prototypes.

**FIGURE 14:**
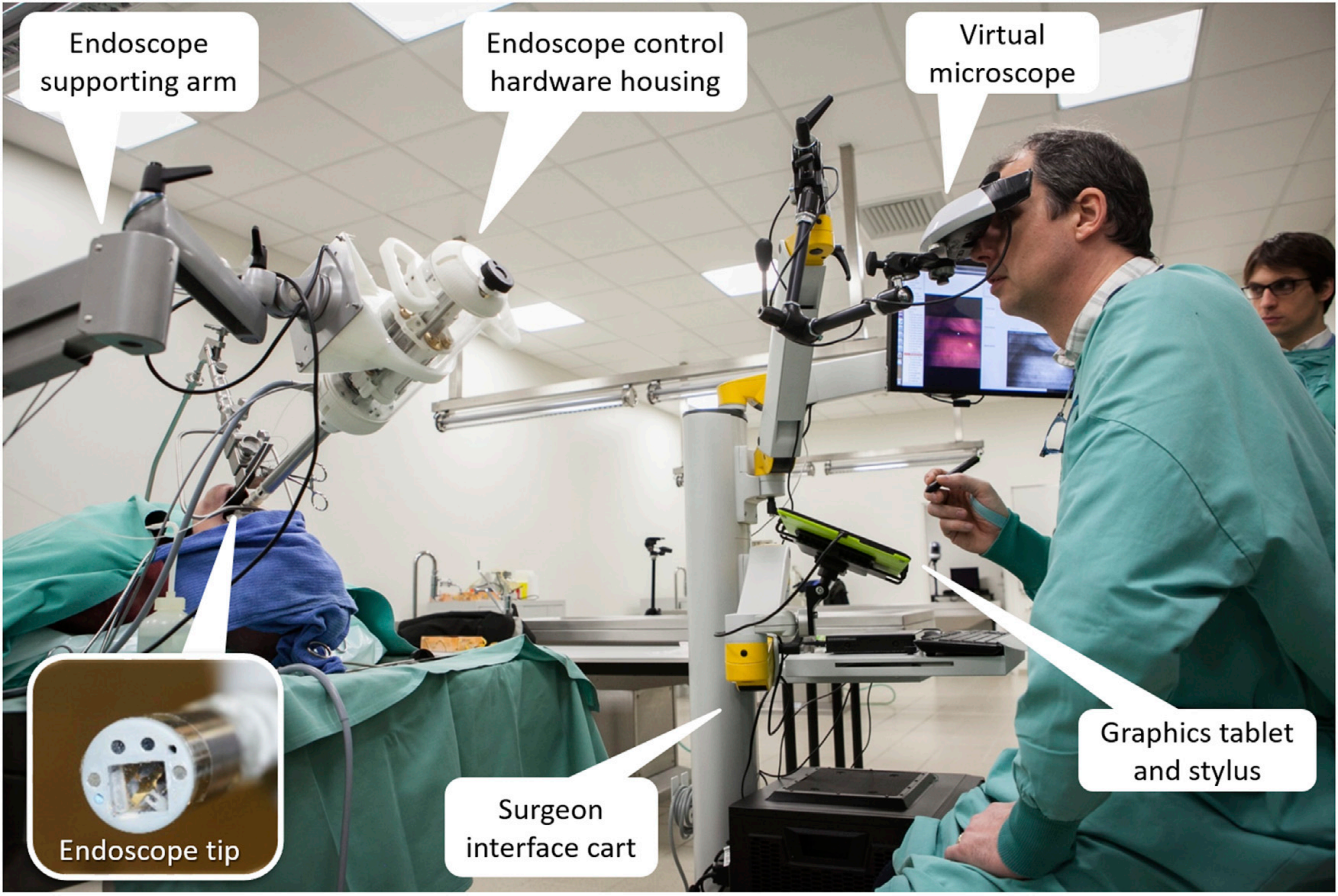
The integrated µRALP surgical system prototype under evaluation in a human cadaver (Tavernier et al., 2017).

The final µRALP system consisted of two main parts: The robotic endoscope and the teleoperation interface, as depicted in Figure 14. The cadaver experiments were instrumental for obtaining performance metrics regarding the complete system and all of its sub-components in a realistic surgical scenario. This experience highlighted the benefits of an integrated solution for robot-assisted endoscopic laser microsurgery, with each system component contributing to enhance surgical precision and quality. It also allowed the identification of system limitations and the acquisition of important clinical feedback, which guided the research and development of the technologies beyond the end of the project as detailed above.

## Discussion

This paper reviewed recent technological advancements and scientific contributions towards robot-assisted endoscopic laser microsurgery, achieved both within and beyond the European project μRALP. Endoscopic laser microsurgeries are challenging and delicate operations that currently lack proper technological support. Consequently, the common goal of the systems reviewed in this paper is the enhancement of current levels of accessibility, precision and quality in such endoscopic procedures. The range of technologies developed for this include flexible robotic endoscopes, micro-mechatronic robotic systems for laser steering, cancer tissue visualization technologies, surgeon-robot interfaces, stereoscopic methods for enhanced teleoperation and automatic control, and cognitive surgical systems. Individually, each of these technologies proved to bring incremental levels of improvement to laser microsurgeries. However, major impact is expected to come from their full integration into a complete clinical surgical robotic system, as preliminarily experienced during cadaver trials with the final μRALP prototype.

At this current point in time, research continues towards improving the TRL of endoscopic laser microsurgery technologies. Despite the remarkable advancements, it is clear from this review that such technologies are still not ready for clinical use. Nonetheless, the critical characteristics and capabilities that endoscopic systems should have to enable effective and high quality laser microsurgeries have been identified.

A flexible and slim endoscope is in general preferred to accommodate for a wide range of patients and to allow inspecting and maneuvering around complex anatomies such as the larynx. The endoscope must integrate illumination and high-definition imaging systems to allow proper examination of tissues and the definition of surgical margins. In addition, it must include the means to deliver a high-power surgical laser to the surgical field. Finally, to facilitate the execution of precise laser ablations, it is beneficial to include also laser focusing capabilities into the system. Continuum endoscopic robots with large central lumen, adjustable deflection, and tip extension capabilities have demonstrated to be a promising solution for these needs.

However, continuum endoscopic robots alone may not answer all needs regarding precise laser steering, high quality laser ablations and high system usability, as the laser beam motions are coupled to the vision system. Integrating a decoupled way to steer the laser beam at the tip of the endoscope would be preferred. This could allow the execution of fast laser scanning motions, which are known to significantly improve ablation quality in soft tissues. For addressing this need, mainly two classes of micro-robotic laser steering systems have been proposed in the literature: one based on actuated mirrors, and the other based on actuated laser fibers. Their specific applicability and utility depend on the intended clinical application and on requirements regarding laser working distance and workspace. Mirror-based micro-mechatronic systems tend to provide the largest laser workspaces, but their miniaturization is challenging. Actuated laser fibers have potential to be made significantly smaller, but the typically drawback is reduced laser scanning range. However, promising solutions to extend the workspace of actuated laser fibers have been proposed and reviewed in this paper.

Apart from size and workspace, also laser scanning speed and accuracy are major requirements to be considered when designing micro-robotic laser steering systems for high precision and high quality microsurgeries. Also here research results have demonstrated that both mirror and fiber-based systems can satisfy these requirements.

The issues that remain open in micro-robotic laser steering include the integration of adjustable focusing capabilities, which may come from coordinated motions with the robotic endoscope, or with the integration of miniaturized active focusing systems. In addition, most systems proposed in the literature still need to address the challenges of integration with a high power surgical laser to demonstrate their expected laser ablation performances. Finally, all endoscopic laser microsurgery systems proposed so far still lack physical tissue manipulation capabilities. This is a critical issue to be addressed to make such systems clinically useful, as tissue manipulation is required for most microsurgical procedures. Technologies from related research areas may be useful here, e.g., concentric tube or other types of miniaturized continuum flexible robots may be used for tissue manipulation, but their integration with the laser microsurgical endoscopes still has to be demonstrated.

Regarding the surgeon-robot interface, research has demonstrated that stylus and tablet-like devices provide a highly intuitive and effective way for controlling the laser beam motion in two dimensions, leading to sizable improvements in terms of surgical precision and safety. This configuration is especially useful when the laser steering system has a relatively long working distance, such as in the case of the mirror-based systems or actuated laser fibers with focusing optics. However, when control of more degrees of freedom is required, other pen-like interfaces (such as the Sensable Phantom Omni or the Force Dimension Omega.6) provide similar usability and laser control performance.

Research results have also shown that stereoscopic visualization of the surgical field is preferred for delicate microsurgeries, as this provides intuitive depth perception to the surgeon. In addition, it allows the integration of advanced 3D vision and automatic control methods to the surgical system, which can enable large improvements in surgical robot control, intraoperative planning and in the quality of laser ablations through adaptive laser focusing.

Computer vision and cognitive systems also have potential for enabling sizable contributions towards enhanced laser microsurgeries. Research reviewed herein has indicated such systems can assist in tumor detection, characterization and identification of margins for conservative tissue excisions. In addition, precise laser incision depth control and volumetric ablation of soft tissues have been demonstrated by these systems, both of which can have direct impact on laser microsurgery precision, safety and efficiency.

Overall, before these technologies can reach clinical use, they still need to prove their safety, robustness, biocompatibility and sterilizability. Cadaver studies can provide critical feedback in this regard, as the µRALP experience has demonstrated. Cadavers offer a realistic surgical scenario while avoiding the ethics issues of animal trials. They can also enable acquiring enough evidence to support the subsequent start of clinical trials, which are required for finally obtaining the certifications for regular clinical use of the new technologies.

Although the road to clinical robot-assisted endoscopic laser microsurgery seems long, it is clear the technologies have potential to bring many benefits to patients, surgeons, hospitals, and the public healthcare system in general. For example, once they reach clinical use, more patients will qualify for transoral laser microsurgeries and benefit from enhanced surgical precision and quality compared to the current state of the art. Surgeons will benefit from a more intuitive surgical setup and from robotic assistance, which will enable them to better plan and execute delicate interventions with reduced surgical trauma. Finally, hospitals will see less complications and revision surgeries, increasing customer satisfaction and, at the same time, contributing to lower healthcare costs.

It is also clear that the new knowledge and the new technologies described herein are applicable to a wide range of microsurgery interventions, both laser and otherwise. This expands the impact of the reviewed research beyond the specific application in robot-assisted endoscopic laser microsurgery.

## Footnotes

1
https://www.olympus-global.com/news/2020/contents/nr01857/nr01857_00001.pdf

2
https://www.medtronic.com/us-en/c/digestive-gastrointestinal/gi-genius.html
